# Supplementary material for: Vitamin D Modified DSS-Induced Colitis in Mice via STING Signaling Pathway
Source: Biology (Basel). 2025 Jun 18;14(6):715. doi: 10.3390/biology14060715 (PMC12190092; doi:10.3390/biology14060715)
Supplement: Supplementary file 1 [file biology-14-00715-s001.zip › Supplementary Materials S1: 16s-report of gut microbiota/Groups/taxanomyBar/taxon_hist/include_all/top10/Genus.groups.distribution.pdf]

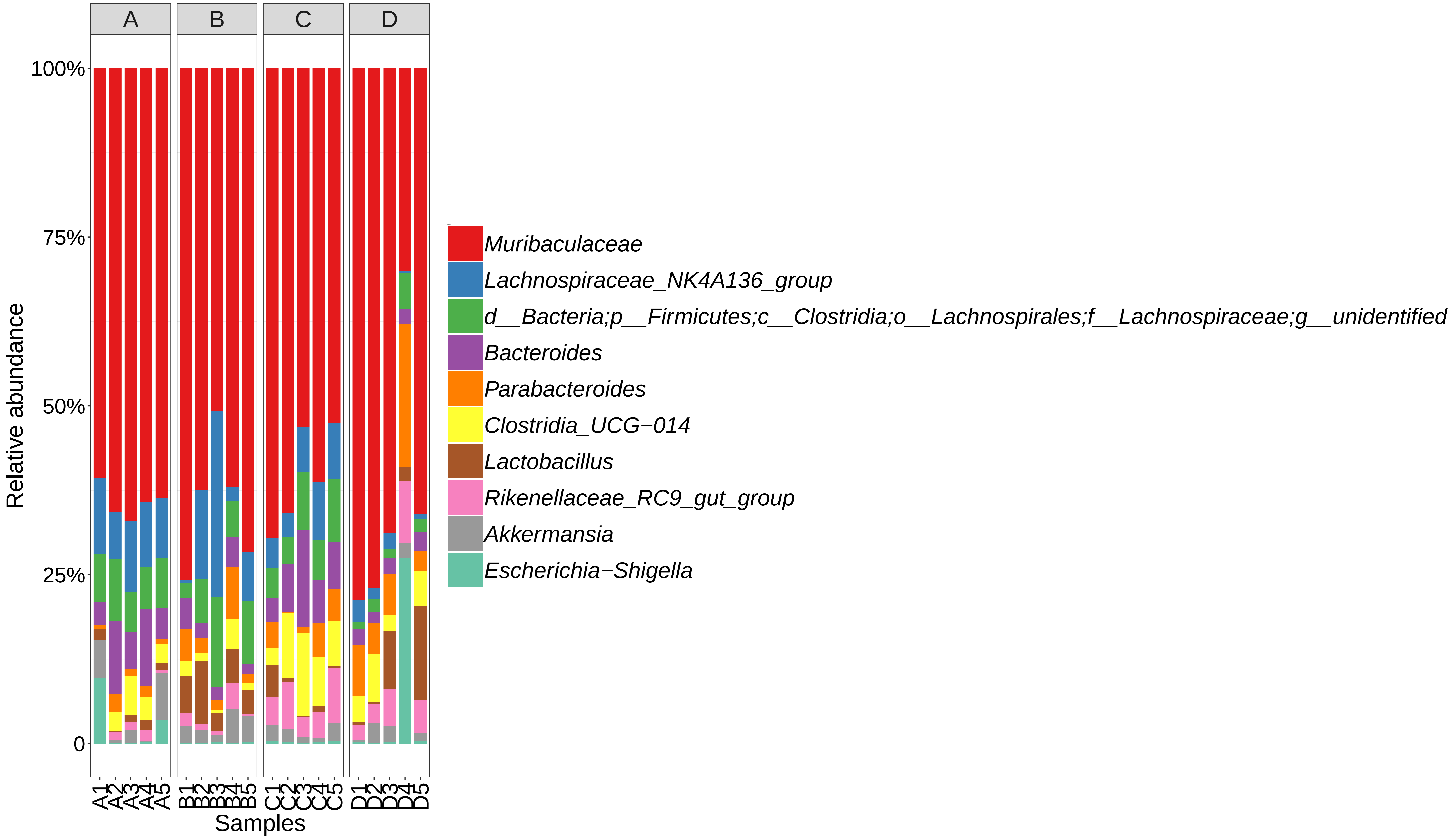
A
B
C
D
100%
75%
50%
25%
0
Relative abundance
A1 A2 A3 A4 A5
B1 B2 B3 B4 B5
C1 C2 C3 C4 C5
D1 D2 D3 D4 D5
Samples
Muribaculaceae
Lachnospiraceae_NK4A136_group
d__Bacteria;p__Firmicutes;c__Clostridia;o__Lachnospirales;f__Lachnospiraceae;g__unidentified
Bacteroides
Parabacteroides
Clostridia_UCG−014
Lactobacillus
Rikenellaceae_RC9_gut_group
Akkermansia
Escherichia−Shigella
